# Regulatory RNAs: role as scaffolds assembling protein complexes and their epigenetic deregulation

**DOI:** 10.37349/etat.2024.00252

**Published:** 2024-07-22

**Authors:** Palmiro Poltronieri

**Affiliations:** Chinese Peoples Liberat Army China (PLA) General Hospital, China; Agrofood Department, National Research Council, CNR-ISPA, 73100 Lecce, Italy

**Keywords:** DNA methylation, RNA modifications, histone marks, non-coding RNAs, scaffolding RNAs

## Abstract

Recently, new data have been added to the interaction between non-coding RNAs (ncRNAs) and epigenetic machinery. Epigenetics includes enzymes involved in DNA methylation, histone modifications, and RNA modifications, and mechanisms underlying chromatin structure, repressive states, and active states operating in transcription. The main focus is on long ncRNAs (lncRNAs) acting as scaffolds to assemble protein complexes. This review does not cover RNA’s role in sponging microRNAs, or decoy functions. Several lncRNAs were shown to regulate chromatin activation and repression by interacting with Polycomb repressive complexes and mixed-lineage leukemia (MLL) activating complexes. Various groups reported on enhancer of zeste homolog 2 (EZH2) interactions with regulatory RNAs. Knowledge of the function of these complexes opens the perspective to develop new therapeutics for cancer treatment. Lastly, the interplay between lncRNAs and epitranscriptomic modifications in cancers paves the way for new targets in cancer therapy. The approach to inhibit lncRNAs interaction with protein complexes and perspective to regulate epitrascriptomics-regulated RNAs may bring new compounds as therapeuticals in various types of cancer.

## Introduction

Epigenetics refers to the modifications of proteins, DNA, and RNA that determine whether a gene is turned on or off. Enzymes involved in these post-translational modifications of DNA, RNA, and proteins, especially histones, called epigenetic marks, are named writers, players, and erasers. Epigenetic mechanisms regulate cell functions, chromatin access, transcription, translation, mRNA splicing, transcript stability, RNA folding, and interaction of RNA with enzymes involved in chromatin modifying complexes [1–5]. In the studies of gene deregulation in cancer, in addition to the great role assigned to oncogenes and tumor suppressors in tumor development, many mutations, deletions, or amplifications are found in genes that belong to epigenetic complexes regulating gene accessibility and gene expression. In addition, a further layer of complexity is provided by non-coding RNAs (ncRNAs). Two main groups of ncRNAs are known, small RNAs: microRNAs, tiRNAs (tRNA derived, stress induced RNAs), and piwiRNAs, in the range of 20–22 bases [3–7], and small nucleolar RNAs (with sizes between 90 and 120 bases), and RNAs longer than 500 bp, that are the focus of this paper: in particular, long intergenic ncRNAs (lincRNAs) involved in cancer will be discussed extensively [8, 9].

In this review, the topic of microRNAs regulating or inhibiting mRNA expression will not be covered, as well as the sponging of miRNAs by long ncRNAs (lncRNAs), since these events occur at the transcriptomic level. Neither the role of lncRNAs in stoichiometric saturation of a target, such as protection of mRNA untranslated regions (UTRs) from miRNA binding, nor stabilization of protein target by interference with ubiquitin ligases, will be discussed. This review intends to focus on regulatory RNAs at the transcriptional and post-transcriptional level, the involvement of lincRNAs as scaffolds assembling protein complexes with a role in chromatin control, their deregulation, and the role of epitranscriptomics in changing the stability, downregulation or upregulation of lncRNAs, that have been reported changed in various types of cancer. Approaches in therapeutic interventions to treat these deregulated cellular functions will be presented.

## Epigenetic enzymes controlling chromatin states

### Methylation of DNA, RNA, and Deamination of RNA

DNA methyltransferases (DNMTs) methylate cytosines in CpG regions using the methyl group donor *S*-adenosylmethionine (SAM) to produce 5-methylcytosine (5-mC/m5C) [1]. DNA methylation transfers a methyl group to cytosine at the C5 position, forming 5-mC in CpG islands (CGIs) with a high G/C content, in proximity to gene promoters [10]. De novo DNA methyltransferases DNMT3A and DNMT3B are responsible for methylation of unmethylated CpG regions during embryogenesis. In adult cells, DNMT1 methyltransferase is required for post-replicative transmission of DNA methylation [1]. Gene expression is regulated by DNA methylation depending on various chromatin proteins and specific transcription factors allowed to bind or not to DNA promoter regions. Methylation of gene promoters by DNMT1 renders the gene marked as not to be transcribed. Chromatin states are classified as euchromatin (open, loosened state, rich in active genes that can be transcribed) and heterochromatin (a more compact state, in which genes are not accessible to transcriptional machinery). Considering the tridimensional disposition in the nucleus, euchromatin localizes to the central part of nuclei, while heterochromatin occupies the peripheral regions of nuclei. Epigenetic changes in nucleotides (RNA and DNA) include cytosine methylation (m5C), and adenine methylation [N6-methyladenosine (m6A)] [11]. NOL1/NOP2/SUN domain (NSUN) family proteins (NSUN1-6) and DNMT2 have been reported as writers of the m5C mark on lncRNAs: readers of this mark are YT521-B homology domain family 2 (YTHDF2), Aly/REF export factor (ALYREF) and Y-box-binding protein 1 (YBX1), and the removal is catalyzed by ten-eleven translocation (TETs; TE1/2/3) and AlkB homolog 1 (ALKBH1) [12]: 5-hydroxymethylcytosine (5hmC) modification reverts the switch-off state into a switch-on state of genes [11].

Other marks on RNA include m6A, m1A, m5C, and 7-methylguanosine (m7G). The methyltransferase-like 3 (METTL3) and METTL14 associate with Wilms’ tumor 1-associated protein (WTAP) to form the nuclear m6A methyltransferase complex (MTC) to methylate adenine to m6A. A second m6A MTC includes RNA-binding motif protein 15 (RBM15), Vir-like m6A methyltransferase associated (VIRMA), zinc finger CCHC-type containing 4 (ZCCHC4), and zinc finger CCCH-type containing 13 (ZC3H13).

METTL3 enzyme relies on methyl group donor SAM. Heterochromatin protein 2 (HP2) chromo domain proteins influence the levels of SAM by CpG methylation of the methionine adenosyl transferase 2A (*MAT2A*) gene.

Among m6A readers are insulin growth factor 2 mRNA binding protein (IGF2BP), heterogeneous nuclear ribonucleoproteins (hnRNPs)—hnRNPA2B1 and hnRNPC, that individuate the target transcripts by recognizing the RBMs accessible through the m6A marks. Other reader proteins are YTH family proteins and eukaryotic initiation factor (eIF).

The fat mass- and obesity-associated protein (FTO) and the ALKBH5 are RNA demethylase enzymes that revert the post-translational methylation of adenine. 2-hydroxy ketoglutarate (2HG) inhibits the activity of FTO and of α-ketoglutarate (α-KG) dependent ALKBH5 N6-methyladenine demethylases. The binding of m6A modified mRNAs to YTHDF2 induces the delocalization of mRNA to the mRNA decay pathway [13].

Apolipoprotein B mRNA editing catalytic polypeptide-like (APOBEC) protein family deaminates cytosine residues in DNA and RNA. Activation induced deaminase (AID) affects DNA demethylation to induce an undifferentiated state [1, 14]. In diffuse large B-cell lymphoma (DLBCL) cell lines, AID cooperates with the TET enzyme TET2 to demethylate Fanconi anemia complementation group A (*FANCA*), increasing its expression [15]. Fumarate and succinate, for their structural similarity to α-KG, may inhibit α-KG dependent enzymes, and regulate TET dioxygenases [1].

### Histone marks

Histone tails are targeted by various post-translational modifications. The activation marks are mono-methylation on histone 3 lysine 4 (H3K4me1), H3K4me2, H3K36me2, H3K4me3, H3K36me3, whereas H3K9me3, H4K20me3, and H3K27me3 are considered repressive [16, 17]. Acetylation and phosphorylation of histone tails decrease the positive charge of histones, disrupting electrostatic interactions between histones and DNA. This leads to a more relaxed chromatin structure, enhancing DNA accessibility for protein complexes and resulting in an increase in gene expression.

Polycomb group (PcG) complexes include Polycomb repressive complexes 1 (PRC1) PRC2 and the recently identified Pho-repressive complex/repressive deubiquitinase Polycomb. PRC1 monoubiquitylates a histone H2A lysine at position 119 (H2AK119ub1) and leads to compaction of the chromatin structure. The presence of hypomethylated CGIs directs the assembly of the PRC2 complex at the target locations. PRC2 has lysine methyltransferase (KMT) activity that H3K27me3: deposition of H3K27me3 marks leads to heterochromatin formation and gene repression. PRC2 consists of several subunits, such as embryonic ectoderm development (EED), suppressor of zeste 12 (SUZ12), and the KMT enzymes enhancer of zeste homolog 1 (EZH1) or EZH2, which activity relies on the catalytic domain SET. PRC2 functions are based on EZH2, EED, and SUZ12 components, in addition to other accessory proteins. EZH2 catalyzes the H3K27me3 mark, a tri-methylated lysine at position 27 on histone 3, a mark that results in transcriptional inhibition of the gene on which the histone is positioned [1]. RNA binding has been shown to be an important regulatory factor in the function of several histone KMTs (HKMTs), such as human EZH2. During the nucleation of heterochromatin, the PRC2 complex includes accessory proteins such as Jumonji, AT rich interactive domain 2 (JARID2), and RNAs. PRC2 associated with accessory, non-core subunits of PRC2 complex, or other cell-type-dependent factors, define nucleation sites, in which H3K27me2 residues become trimethylated to H3K27me3. PRC complexes recruit DNMT enzymes to interact with these chromatin regions and render them not accessible to transcription complexes [18]. These interactions involve also ncRNAs, either as binding partners for DNMT as well as for PRC subunits [19].

The protein complex performing an antagonistic activity with respect to PRC is called the chromatin activating complex TrxG and the principal component is Trithorax in drosophila (related to brahma in the SWI/SNF chromatin remodeling complex), the homolog mixed-lineage leukemia 1 (MLL1) in humans, and absent, small or homeotic (ASH1), whose SET domains perform histone H3K4 methylation [1].

#### Writers of lysine methylation

HKMTs or PKDMs control gene expression programs through the PTM of lysine in histone tails at specific positions. Protein KMTs (PKMTs) contain a characteristic SET domain. The SET domain consists of two, SET-N and SET-C globular regions at N- and C-terminal ends. Each region contains three-to-four short β-strands, a short helix, and various loops connecting these structural motifs. SET-C forms a topologically unusual knot-like structure in which a strand threads through a loop region. The residues that form the knot are the most highly conserved in SET proteins. The amine oxidase family includes lysine-specific demethylase 1 (LSD1; KDM1A) and LSD2 (KDM1B), using flavin adenine dinucleotide (FAD) as cofactor, and are involved in demethylation of mono- and dimethylated lysines, but not trimethylated lysine. The Jumonji-C (JMJC) domain family uses a Fe^2+^- and 2-oxoglutarate-dependent dioxygenase mechanism, and is able to demethylate mono-, di-, and trimethylated states. The histone-lysine *N*-methyltransferase 2 (KMT2) family (MLL1, MLL2, MLL3, MLL4, SETD1A, and SETD1B) exerts histone-lysine methylation when associated with COMPASS, a large multi-subunit protein complex that catalyzes di- and tri-methylation of histone H3 lysine at positions 4 (H3K4) and regulates for RNA-dependent polymerase II transcription in active compartments of chromatin. SETD2 downregulation was associated with several cancers [20]. The cancer expression of KMTs such as NSD1 is linked to SAM availability and is inversely correlated to the activity of nicotinamide-*N*-methyltransferase (NNMT), whose product, 1-methyl nicotinamide, is a methyl sink for an excess of methyl groups, carried by the methyl donor SAM [21].

On the other side, Dot1 family members, methylating in H3K79, contain conserved sequence motifs characteristic of class I SAM-binding methyltransferases, such as DNMTs and protein arginine methyltransferases (PRMTs).

#### Readers of methylated lysines

Chromodomain, Tudor domain, PWWP domain, PHD domain, and malignant brain tumor (MBT) proteins are methylated lysine (Kme) readers. Chromodomains present in transcriptional silencers, such as the heterochromatin-associated protein HP1, are readers of methyl-lysine histone modifications. HP1α contains a chromodomain that serves as a reader for H3K9me3, while its chromo shadow domain is a writer in association with additional enzyme proteins that produce the H3K9me3 mark, making this enzyme a bifunctional reader/writer. ZMYND11 is a reader of H3K36me3 marks through its PHD-bromo-PWWP (PBP) domain [22, 23] as well as a noncanonical reader of arginine methylation on hnRNPA1, inhibiting its role in stress granule formation [24].

#### Erasers of methylation on lysines

Histone demethylases (HDMs)/KDMs include SET domain containing Suv39h1/Suv39h2, G9a/GLP, amine-oxidase type LSDs and JMJC domain-containing histone demethylases. LSD1 specifically demethylates H3K4me1 and H3K4me2. LSD2 (KDM1B) specifically targets H3K4me2. LSD1 interacts also with chromatin remodeling complex SWI/NSF1 related actin-dependent SMARCA2, also known as Brahma homolog (BRM) [1]. Fumarate, succinate, and α-KG, accumulating during oxidative phosphorylation, are inhibitors of α-KG dependent enzymes, and by co-substrate competition may regulate histone lysine demethylases [25].

#### Writers of lysine acetylation

HATs are histone acetyltransferases that induce a relaxed state in chromatin. Among several isoforms are lysine acetyltransferase KAT2A, KAT2B, KAT5, KAT6A, KAT6B, and KAT8. α-KG upregulates H3/H4 histone acetylation, through increased availability of acetate.

#### Readers of acetylation on lysines

Bromodomains, present in several different transcriptional regulatory proteins, such as the HATs GCN5, p300, and CBP are readers of acetylated lysines. Among readers are bromo- and extra-terminal (BET) domain and bromodomain proteins, that recruit transcriptional regulatory complexes and chromatin remodeling complexes. For example, BRD4, belonging to this group, has been found translocated in several cancers. Tudor domain proteins have also the ability to bind to methyl arginine and are molecular adaptors: the constituents of chromatin remodeling complexes, AT rich interactive 1A (ARID1A) and ARID4A interact with EZH2 and are mutated in hepatocellular carcinoma (HCC), liver, kidney, ovarian and colon cancers.

#### Erasers of acetylation on lysines

KDACs or HDACs are histone deacetylases of various groups, exploiting a zinc ion in the catalytic site. HDACs use nicotinamide adenine dinucleotide (NAD) as substrate to form nicotinamide and acetylated ADPr but perform other reactions, such as de-acylation (demyristoylase, depalmitoylase, and similar reactions). The nucleosome remodeling and deacetylase (NuRD) cooperates with PRC to induce repression of chromatin [26].

#### Writers of arginine methylation

Arginine methylation is regulated by nine PRMTs, subdivided into three classes based on the type of methylation. The type I PRMTs (PRMT1, PRMT2, PRMT3, PRMT4, PRMT6, PRMT8) generate asymmetric dimethylarginine; type II PRMTs (PRMT5 and PRMT7) form symmetric dimethylarginine (SDMA); type III PRMTs (PRMT7) form monomethylarginine, while PRMT9 does not resemble any of these classes. PRMTs are involved in mRNA splicing, X chromosome inactivation, DNA repair, and tumorigenesis.

#### Interactions of PRC enzymes, DNMTs, and chromatin remodeling complexes

In colon cancer, scientists found that DNA hypermethylation is associated with interaction between DNMT3B, PRC1, and PRC2, as well as other chromatin complexes [18]. These epigenetic marks-modifying complexes are associated with secondary proteins as well as RNAs [27, 28].

## RNAs regulation at the transcriptional and post-transcriptional level

The first types of RNA that were studied were ribosomal RNAs, transfer RNAs, microRNAs, and snoRNAs. Software assists researchers that study RNAs with analysis of RNA secondary structures, such as RFAM, QRNA, RNAz, RNAfold, Evofold, and Foldalign. Stable RNA modifications (polyA tails) are required for the activity of the RNA in the cytoplasm. Other modifications on RNA are tightly regulated by writers, readers, and erasers: RNAs may be processed by enzymes epigenetically, such as deaminases and methylating enzymes [29, 30]. These modifications can alter the base pairing and reduce the affinity towards a complementary sequence, or alter the masking of a target site on proteins or mRNAs.

In the middle of 2005, the first findings were published showing that a part of the transcribed RNAs were indeed functional [31]. At the same time, studies started to define the RNA world in a high-throughput format, such as siRNA libraries for silencing many ncRNAs in cell cultures in microplates, chromatin immunoprecipitation (ChIP) methods combined with enrichment of transcribed RNAs [ChIP; RNA-immunoprecipitation (RNA-IP)] and methods combined with next-generation sequencing (NGS). Three-dimensional motifs in RNA architecture can encode spatial information. For instance, kink-turns (k-turn) are RNA motifs that present both double-stranded and single-stranded components and induce a three-dimensional structure into helical stems [32].

## Regulatory RNAs

lncRNAs are sequences of RNA with sizes from hundreds to thousands of nucleotides, originating from various parts of the genome, from introns, or across gene sequences, or in the proximity of a protein coding gene. These lncRNAs can be polyadenylated or non-polyadenylated. Thus, the compartmentalization can be solely nuclear in the second case, or they can be present in nuclei and in the cytosol. The functions and the information that lncRNAs contain are multiverse, being able to code for small peptides and to originate microRNAs. Regulatory RNAs sum up the largest number of transcripts from genomes [8]. Many of them have been proven to be functional, using CRISPR/Cas technology or RNA silencing.

lncRNAs can block microRNAs by acting as competitive endogenous RNA (ceRNA) and miRNA sponges, preventing miRNA-mRNA binding, thus preserving the mRNAs targeted by miRNAs from degradation [33]. miRNAs, which inhibit mRNA expression by pairing to complementary target sequences on mRNAs, may be sequestered or sponged by lncRNAs for the presence of one or more sites complementary to one or more miRNAs: this phenomenon is known as an interference on the activity of any type of RNA by a ceRNA. In this way, lncRNAs sequester the miRNAs freeing the mRNA targets from inhibition and degradation. Their functions are numerous, either at the transcriptional as well as a post-transcriptional level. Due to their involvement in cell proliferation, activation of oncogenic signals by means of overexpression or higher stability, or on the opposite by their ability to block oncogenic signaling, as by their downregulation in tumors, regulatory RNAs, similarly to miRNAs, have been classified as oncogenes or as tumor suppressors. In a specific cell type setting, they may behave as oncogenes, while in another cell type may act as suppressors; this is due to the presence of several combinations of domains in a lncRNA, enabling them to act as miRNA sponges, and at the same time to code for a peptide, or to interact with epigenetic regulatory mechanisms. The main functions described up to now are the following: lncRNAs can regulate transcription by acting as a scaffold by binding proteins together in a complex structure. A second function of a lncRNA is as a guide of proteins or other molecules to target a genomic location, or as a guardian to protect the binding site from post-transcriptional modifications or interactions with other proteins: lncRNAs, by binding to their targets, have been involved in stabilization of proteins by interference with ubiquitin ligases, and in protection of RNA and protein targets from downstream processing. LncRNAs can directly bind to genomic regions within the genome to transduce signal activation of DNA-bound molecules. LncRNAs function as scaffolds to assemble protein complexes in chromatin regulation, but also in other cellular signaling, such as DNA damage response (DDR). *GUARDIN* (long non-coding transcriptional activator of miR34a) is activated by p53 following DNA damage. In the nucleus, *GUARDIN* functions as an interaction partner that binds to breast cancer type 1 susceptibility protein (BRCA1) homologue and to BRCA1 associated RING domain 1 (BARD1), and the formation of the protein complex allows the recruitment of DNA double-strand break (DSB) repair machinery [34].

In addition, lncRNAs function as decoys, preventing different proteins from binding to targeted genomic regions. p21-associated ncRNA DNA damage-activated (*PANDA*) is a decoy for nuclear transcription factor Y subunit-α (NF-YA), thus sequestering it from promoters of target genes, producing a reduction in apoptosis and cell senescence [35]. In addition, some lncRNA is also transcribed, forming short peptides or micropeptides that have functional roles. In this review, the topic of microRNAs regulating or inhibiting mRNA expression will not be further discussed, as well as the sponging of miRNAs by ncRNAs. This double-edged character of many lncRNAs is relevant for the different effects in different cell types so that an RNA can behave as oncogene or as antioncogene depending on the role of microRNAs in that cell. In addition, it can produce important effects when therapeutic interventions are studied, since silencing one lncRNA may have consequences also on its ability to bind microRNAs.

Interestingly, numerous functions assigned to regulatory RNAs deal with epigenetic regulation. This review will focus on lncRNAs with nuclear localization that are active transcriptionally and post-transcriptionally (epitranscriptome) and interact with epigenetic factors [36–39]. A large group of lncRNAs control the transcription of genes in genomic loci close to their genomic position, either acting as enhancers or interacting with enhancers of target genes. For the post-transcriptional activity, it occurs with various mechanisms, such as the binding to regions of mRNAs or by interactions with splicing factors and with RNA modification enzymes.

Considering the most debated role of these RNAs, the scaffolding ability to bring together proteins, such as the epigenetic modifiers, several studies have provided no enough evidence of the binding to the supposed interacting proteins. While the studies on short sequences of a specific RNA may be informative on the minimal region required for interaction with proteins, the full-length contains sequences additive in the binding to one or more protein partners, for example by changing the orientation of the hairpins, or the overall secondary structure. This may set the basis for discordant results on the interactions between lncRNA with protein partners, obtained using short sequences of RNAs with respect to full-length sequences.

Furthermore, while immune studies such as western blots preserve antibody specificity and do not show cross-reactions with other proteins, the RNA-IP protocols involving chemical crosslinks such as the use of formaldehyde, have shown that anti-EZH2 antibodies cross-reacted with an RNA binding protein (RBP), making the results of these experiments not specific for EZH2 binding. There is a need for unequivocal IP protocols and confirmation of direct interaction between RNAs and target proteins to validate the results of RNA-IP [40]. The data obtained by RNA-IP from formaldehyde-crosslinked ESCs have produced a too variable and large number of lncRNA binding to EZH2, but the specificity of the interaction has been invalidated by cross-reactive antibodies [40]. On the other hand, observation of interaction between RBPs and RNAs is feasible using North-western methods. Direct methods for analysis of protein complexes-RNA or protein-protein interactions to confirm the results are required. In the following paragraphs, after discussing the role of lncRNAs in cancer development, a special focus will be put on riboregulators acting in concert with other proteins, either with RNA binding ability or with preferential affinity for three dimensional structures in RNA, and for this ability defined as scaffolding RNAs.

## RNAs acting through PRC

A group of lncRNAs are scaffolding RNAs, with a function in tethering to protein complexes involved in epigenetic regulation of histone marks, chromatin, and gene methylation: they present complex tridimensional structures, able to work as scaffolds and assemble and guide protein complexes and enzymes involved in epigenetic modifications to specific gene loci and final targets.

Long interspersed nuclear elements-1s (L1s) are transposable elements acting as lncRNAs that regulate cortical development by associating with chromatin, and interacting with the PcG proteins EZH2 and SUZ12, to deposit H3K27me3 histone marks, orchestrating the regulated gene expression during brain development [41, 42].

One of the first examples of an RNA acting as a scaffold for proteins is HOX transcript antisense intergenic RNA (HOTAIR) [43]. This lncRNA can recruit chromatin-modifying complexes and is involved in the repression of the distal *HOXD* genes. Other activities were additionally assigned to these lncRNA. HOTAIR knockdown suppressed the expression of NLRP3, pro-caspase-1, and pro-IL-1β, as well as IL-1β maturation and pyroptosis, avoiding NLRP3 inflammasome activation, and blocked ROS generation induced by high glucose. Moreover, HOTAIR binding and inactivating Nrf2 promoted Nrf2 and Keap1 interaction [44].

This lncRNA regulates both histone methylation and acetylation. In exosomes, it promotes proliferation, invasion, and migration and inhibits the apoptosis of endometrial stromal cells. HOTAIR induces the switching of histone H3K27 acetylation to methylation in the E cadherin promoter, which induces inhibition of E-cadherin transcription, promoting epithelial-to-mesenchymal transition (EMT) in gastric cancer. In cancer stem cells of the liver, HOTAIR blocked the recruitment of CREB, P300, and RNA polymerase II (Pol II) to the SETD2 promoter region [45, 46].

Pull-down experiments showed the association of purified human PRC2 with HOTAIR RNA as well as with other RNAs [47–50]. Wu et al. [51] showed that the PRC heterodimer EZH2-EED was required for binding to HOTAIR, which was proposed to recruit SUZ12 and EZH2 subunits, to attract PcG repressing complexes (PRC1, PRC2) mediating the histone repressing mark (H3K27me3) in different genes. HOTAIR was described as a regulatory RNA that binds to EED-EZH2-SUZ12 subunits of PRC2 complexes, inducing epigenetic gene silencing. HOTAIR also recruits histone demethylase complexes, with LSD1 (KDM1A) to the H3K4me mark.

However, several recent reports [27, 28, 48] proved there is not a direct involvement between EZH2 and HOTAIR. Assuming that EZH2 is not directly bound to HOTAIR, there must be other epigenetic interactive proteins, possibly with RNA binding activity, or by the aid of a bridging RNA [52] or a bridging protein such as JARID2 [49]. Kanhere et al. [53] studied RNA binding by HOTAIR using as probes EZH2, SUZ12, EED, and RBBP4 and confirmed RNA binding to SUZ12 [54]. RNA binding was not detected for any other PRC2 subunit, including EZH2. Phosphorylation sites in EZH2 may be relevant for interaction with lncRNA, as well as for EZH2 binding to protein partners (site T345 in mouse and T350 in human sequence for HOTAIR, and T487 in SUZ2 binding domain in mouse sequence) [48]. PRC2 protein EZH2 was shown to interact with DNMTs and supports the localization of DNMTs to specific loci [55].

Portoso et al. [56] showed that PRC2 is dispensable for HOTAIR dependent decrease in transcription and in changes in methylation levels of histone 3. HOTAIR was shown to be dispensable to bind PRC2, and the binding was shown neither specific nor strong. HOTAIR was described as a tethering RNA bringing in close proximity two enzyme complexes [55], the first one formed by EE2-SUZ12-EZH2, the second one formed by RE1-silencing transcription factor (REST) complex LSD1-REST-CoREST that leads to H3K4 demethylation, affecting gene silencing in cooperation with H3K27me3.

When *HOTAIR* RNA was overexpressed in MDA‐MB‐231 breast cancer cells, either in the presence or absence of PRC2, few transcriptomic changes were detected [56]. Chromatin changes have been observed even in the absence of EZH2, showing that repressive activity may be linked to chromatin rearrangements and histone demethylation by LSD1 or by other yet unknown remodeling factors [57].

It is known that topologically associating domains (TADs) and compartments define the arrangements of heterochromatin regions on the periphery of nuclei, and active transcribed regions located in the central regions of nuclei, associated together by loops. The nuclear pore components (Nup proteins, cohesins, lamins) are implicated in the control of active and inactive genomic regions, and in the formation of TAD and compartments relegating heterochromatin at the nuclear periphery and euchromatin regions in the active center of nuclei, looped by organizing and assembling complexes [58]. Among the additional roles assigned to HOTAIR, this lincRNA can interact with E3 ubiquitin ligases and their substrates, which enhances the ubiquitination and degradation of Ataxin‐1 and Snurportin‐1 [59].

However, up to now the direct involvement of HOTAIR in EZH2-dependent gene silencing has not been demonstrated. One possible hypothesis is an indirect role, mediated by a second RNA, bridging HOTAIR to EZH2 or one of its subunits. A second possibility is that HOTAIR-dependent H3K27 methylation proceeds independently from EZH2-dependent H3K27 methylation, and both are additive in suppressing gene expression.

The initial studies on EZH2 interaction with RNAs identified a series of hundreds of RNAs that were pulled down by RIP-seq and ChIP-seq, however, it seemed a number too high, exceeding the numbers of RNAs really able to interact [60]. Wang et al. [61] analyzed 1,328 lncRNAs with EZH2 affinity, discussing 858 RNAs related to specific cancer and others found overexpressed in several cancer types. They proposed a common motif with paired two 4-nt loop secondary structures as a feature responsible for the interaction with PRC2. However, the affinity for EZH2 binding by short fragments of RNAs has been put in discussion, since the specificity seems to increase of only a few folds when challenging EZH2 with short or long RNA sequences, while a complete sequence may assume a structure interacting with the protein in vitro and in vivo. Davidovich and Cech [48] therefore proposed a few possible explanations for the progressive steps leading to binding and interaction. After extensive experiments, Long et al. [27, 28] published evidence supporting the requirement of RNA as a constitutive element for PRC2 to bind to chromatin regions and to exert H3K27me3 marks for repression. It is necessary to note that many publications discussed the involvement of regulatory RNA and the downregulation of target genes presenting insufficient evidence of interaction with EZH2. The sentence “mechanistically, RNA interacted directly with EZH2 and induced H3K27 methylation to silence target genes” cannot be accepted without confirmation experiments, but it may be presented as “possibly, the interaction may be indirect, or mediated by other RNAs, or mediated by proteins bridging the RNA with EZH2”.

Using the RIP-seq method, Ye et al. [60] identified 94 lncRNAs associated with EZH2 in neuroblastoma, among which small nucleolar RNA host gene 7 (SNHG7), SNHG22, KTN-AS1 and Linc0084 were identified.

For instance, several lncRNAs are involved in the maintenance of pluripotency in cardiac embryonic cells. HBL1, derived from the *LINC00458* (also known as lncRNA-ES3 or ES3) gene, forms the last exon of some LINC00458 splice isoforms. HBL1 is necessary for PRC2 occupancy at genomic regions and guides PRC2 function to pose H3K27me3 marks during early human cardiogenesis [62, 63].

Furthermore, HOTAIR is also involved in gene activation of downstream genes. HOTAIR, expressed at high levels, interacts with the androgen receptor (AR) in renal cell carcinoma and cooperatively promotes GLI2 transcription by binding to its promoter. High GLI2 levels activate the Hedgehog signaling pathway [64]. ZFAT-AS1 inhibited CDX2 transcription by interacting with PRC2 in glioma and promoting the deposition of H3K27me marks on its promoter [65].

A list of RNAs repressing gene expression through PRC2 recruitment is shown in Table 1.

**Table 1:** RNAs repressing gene expression through PRC2 recruitment

**Gene**	**Partner**	**Target**	**Modification**	**Tumor**	**References**
*LINC01133*	EZH2, LSD1	KLF2, p21, E-cadherin	H3K27me	NSCLC	[66]
*PEG3*	EZH2	Yy1	H3K27me	Endothelium	[67]
*ANRIL-CDKN2B-AS1*	PRC1–CBX7, PRC1	P14arf, p16ink4	H3K27me	-	[68]
*HOXA-AS2*	EZH2, LSD1	P21, KLF2	H3K27me	CRC	[69]
*Linc01088*	EZH2	P21	H3K27me	NSCLC	[70]
*SNHG6*	PRC2	P21, KLF2	H3K27me	Osteosarcoma	[71]
*Linc01088*	EZH2	P21	H3K27me	NSCLC	[70]
*LL22NC03-N64E9.1*	PRC2	KLF2	H3K27me	CRC	[72]
*LINC00702*	EZH2	KLF2	H2K27me	Ovary	[72]
*DUXAP10*	LSD1	LATS2	H3K27me	NSCLC	[72]
*DUXAP10*	EZH2, LSD1	CDK1A, KLF2	H3K27me	Pancreas	[73]
*LINC00665*	EZH2, LSD1	KLF2	H3K27me	Prostate	[73]
*MYLK-AS1*	PRC2	LATS2	H3K27me	Gastric cancer	[74]
*SNHG20*	PRC2	CDKis	H3K27me	NSCLC	[75]
*UCA1*	PRC2	P27	H3K27me	BC	[76]
*SNHG7*	PRC2	CDKis	H3K27me	-	[77]
*SNHG7*	EZH2	CDKi	H3K27me	Ovary	[77, 78]
*TUG1*	PRC2	KLF2	H3K27me	Hepatocell HCC	[78]
*ARHGAP27P1*	PRC2	CDKis	H3K27me	-	[79]
*circRap1b*	KAT2A	HOXA5, Fam3a antiapoptotic factor	Low H3K9ac	Prevents stroke	[80]
*ATRX*	PRC2	Xist targets	H3K27me	Chromatid silencing	[81]
*PVT1*	PRC2	Apoptotic genes	H3K27me	Multiple myeloma	[33]
*XIST*	PRC2, SHARP, HDAC3	RNA Pol II exclusion	histone deacetylation	X chromosome	[82, 83]
*CHASERR, HASTER*	unknown	Transcription factors	-	Various	[84, 44]
*SNHG17*	PRC2	CDKis	H3K27me	Gastric cancer	[44]
*CASC11*	EZH2	PTEN	H3K27me	CRC, HCC	[85]
*FIRRE*	hnRNPU	X chromosome	H3K27me	Set up specific chromosomal domains	[85]
*Xist*	HDAC3, SHARP TRIM28, SMRT	X chromosome	Deacetylation	Silencing, RNA Pol II pausing	[83]
*GIHCG*	EZH2, DNMT1	miR-200b/a/429	H3K27me CpGme	Hepatocell HCC renal cell cancer	[85]
*PVT1*	EZH2	miR-200b	H3K27me	Cervical cancer	[85]
*ILF3-AS, HEIH*	EZH2	miR-200b/a/429	H3K27me	Melanoma	[85]
*NEAT1*	BRG1 SWI/SNF	GADD45A	Silencing	Gastric cancer	[85]
*LINC00628 antioncogene*	EZH2	LRRN2, CCNA2 cell cycle genes	H3K27me3	Downregulated gastric cancer	[85]
*PART1*	EZH2 PLZF	PDGFB	H3K27me3	Gastric cancer	[85]
*LINC01446*	LSD1	RASD1	Suppression by demethylating H3K4	Gastric cancer	[85]
*LINC00673*	LSD1, EZH2	KLF, LATS2	H3K4 demethylation H3K27me3	Gastric cancer	[85]
*LINC01232*	EZH2	KLF2	H3K27me	Gastric cancer	[85]
*LINC00202*	EZH2	KLF2	H3K27me	Gastric cancer	[85]
*PLZF*	EZH2	PDGFB	H3K27me	Gastric cancer	[85]
*LINC00675*	LSD1	SPRY4	H3K4me2 demethylated	Gastric cancer	[85]
*HOXA11-AS*	EZH2, LSD1, DNMT1	KLF2, PRSS8	H3K27me3, H3K4me demethylation	-	[85]
*BST2/BISPR*	-	Tetherin	Positive regulator of BST2/tetherin	Antiviral	[85]
*SNHG12*	HDAC9	miR-320a downregulated	Resistance to doxorubicin carboplatin sunitinib, temozolomide	Ovarian cancer Osteosarcoma	[85]
*SNHG13/DANCR*	KAT6A (H3K23ac), EZH2	Cell cycle inhibitors	-	CRC, endocrine cancers	[85]
*SNHG14*	EZH2, PRC2	-	-	Endocrine-related cancers	[85]
*SNHG15*	EZH2, PRC2	-	-	Endocrine-related cancers	[85]
*lncRNA-CMPK2*	-	ISG, IFN-response block	-	-	[85]
*NRAV*	-	IFN-response block	-	-	[85]
*NRIR*	-	IFN-response block	-	Systemic sclerosis	[85]
*CHROMR*	IRF2BP2	IFN-response active	Sequesters IRF-2/IRF2BP2 repressor complex	Antiviral	[85]
*LncATV*	-	IFN-response block	Restricts RIG-I- mediated immunity	Antiviral	[85]
*LUCAT1*	-	IFN-response block	-	Antiviral	[85]
*LNMAT1*	EZH2	CADM	H3K27me	Melanoma	[85]
*FEZF2-AS*	LSD1	CDKi	H3K4me2 demethylation	Gastric cancer	[86]
*HOTTIP*	CTCF, DNMT1	MTUS1	-	NSCLC	[87]
LINC01271 mamm. tumor associated RNAs (*MaTAR25*)	PUB	Tensin 1 promoter	Upregulates tensin 1 in focal adhesions, impacting migration	CRC, BC	[85]

CASC11: cancer susceptibility 11; EZH2 enhancer of zeste homolog 2; LSD1: lysine-specific demethylase 1; PRC1: Polycomb repressive complexes 1; NSCLC: non small cell lung cancer; CHASERR: CHD2 adjacent suppressive regulatory RNA; PVT1: plasmacytoma variant translocation 1; CBX7: chromobox protein homolog 7; CRC: colorectal cancer; CDKi: cyclin-dependent kinase inhibitor; BC: breast cancer; Xist: X-inactive speciﬁc transcript; CTCF: CCCTC-binding factor; GADD45A: growth arrest and DNA-damage-inducible alpha; PTEN: phosphatase and tensin homolog; -: blank cell

Among genes that control proliferation and cell cycle, a group of CDKi p15, p16, p21, p27, p57, Kip, have been described as silenced by hypermethylation of their promoters, with involvement of different RNAs, often specific to a cell type and type of tumor [65–71].

INK4a/ARF/INK4b locus on chromosome 9p21 gives rise to the CDK inhibitors p15(INK4b), p16(INK4a), p19(CDK4d) and p19/ARF (inhibiting MDM2), which play independent roles in tumor suppression as inhibitors of the cell cycle.

The ARHGAP27P1 lncRNAs are downregulated in gastric cancer, and its silencing allows Jumonji-domain containing 3 (JMJD3) to demethylate H3K27me3, inducing the expression of CDKi p15, p16 and p57 [79].

Several forms of RNAs, such as GClnc1, GCAWKR, and some circular RNA (circRNA) are involved in the recruitment of acetyltransferases such as KAT2A [WD repeat domain 5 (WDR5)/KAT2A complex] to the gene promoter, adding the H3K4me3 and H3K9ac marks in absence of H3K27me3 [80, 88].

Considering a different group of regulatory RNAs, ATRX RNA helicase remodels RepA and Xist RNAs to increase PRC2 binding [81]. Xist is the master regulatory RNA that controls the silencing of one chromosome X in females and is a scaffold for other RNAs and PRC repressive complexes. HKMTs, enzymes that methylate lysines on histones and other proteins, can bind RNA even without a canonical RNA binding domain. An RNA binding region within KMT2D (also known as MLL2, MLL4) allows its binding to an RNA pool, but the different binding specificities depending on RNA substrates suggest the presence of other regulatory elements. KMT2D binds co-transcriptionally to the mRNAs of genes under its control, by interacting with super enhancer-RNAs (eRNAs) and splicing-related ncRNAs [82].

The apoptotic protease-activating factor 1 (APAF1)-binding lncRNA (ABL) promotes proliferation and drug resistance in gastric cancer by blocking apoptosis and caspase activation, by binding to APAF1 and inhibiting the interaction with cytochrome c; intriguingly, encapsulated liposomal siRNAs targeting ABL reestablished sensitivity to chemotherapy [85]. Another possible target is m6A writers acting on ABL, or inhibition of m6A reader IGF2BP1, to destabilize ABL RNA.

LncRNA such as CHASERR, PVT1, and HASTER [also known as hepatocyte nuclear factor 1 homeobox A (HNF1A)-AS1] act as transcription-stabilizing elements that regulate the activity of dosage-sensitive genes encoding transcription factors [44, 83, 84]. Through genetic experiments, deregulation in these transcription stabilizers can cause dramatic phenotypes. Considering lincRNA-p21 [89] and Maenli (Gm29348), it was shown that these RNAs support local activation of gene transcription [90]. In other cases, several lncRNAs can influence gene transcription in trans.

## LncRNAs and gene silencing by CpG methylation

Various lncRNAs interact with genomic loci by organizing RNA-DNA triplex structures at promoters [8]. EZH2 and CTCF interact and bind to the CDKN2B promoter region. An RNA-DNA triplex is formed by CDKN2B-AS1, and then the CDKN2B promoter recruits EZH2 and CTCF [86].

PRC2 interacts with DNMT1, DNMT3A, DNMT3B and DNMT 3-like (DNMT3L). DNMT3L binds to PRC2 in competition with Dnmt3a and Dnmt3b, to regulate the methylation levels at H3K27me3 [48].

A further activity assigned to regulatory RNA is in the assembling of DNMT1 complexes to induce hypermethylation of promoter CGIs in order to silence target genes [11, 33]. In the alternative, an opposite mechanism requires RNA occupancy of promoters avoiding the binding of DNMT1 and modification of CGIs at promoters [91, 92]. DACOR interacts with and inhibits DNA methyltransferase DNMT1 [93, 94].

A series of lncRNAs associated with DNMTs silencing genes by CpG methylation is presented in Table 2.

**Table 2:** LncRNAs associated with DNMTs silencing genes by CpG methylation

**lncRNA**	**recruitment**	**target**	**Mechanism**	**Reference**
ecCEBPA	DNMT1	CEBPA	-	[92]
DACOR	DNMT1	DNMT1	Decoy	[93, 94]
LINC0051	EZH2	PTEN	CpG methylation	[95]
HOTAIR	DNMT1	PTEN	CpG methylation	[96]
SAMD12-AS1	DNMT1	P53	CpG	[97]
KIF9-AS1	DNMT1	RAI2	CpG	[98]
HOTAIR	DNMT1	miR-122	CpG	[99]
TINCR	DNMT1	miR-503	CpG	[100]
AIRN	Steric hindrance	IGFR2R	CpG	[101, 102]
HITT	EZH2, PRC2	Hif-1alpha	Triplex formation	[85]
CDKN2B-AS1	PRC2	CDKN2B	Triplex formation	[84]

ecCEBPA: Extra-coding CEBPA; HOTAIR: HOX transcript antisense intergenic RNA; PTEN: phosphatase and tensin homolog; PRC1: Polycomb repressive complexes 1; -: blank cell

DNMT enzymes make use of methyl donor SAM. SAM is produced by the methionine adenosyltransferase (MAT), starting from ATP and methionine, while *S*-adenosyl homocysteine (SAH) is hydrolyzed by SAH hydrolase (SAHH), acting as a DNMT feedback inhibitor. Regulation by lncRNAs of MAT and SAHH, the genes involved in SAM synthesis or SAH degradation, may lead to malfunctioning of DNMTs. H19 RNA binds to SAHH and inhibits its function, thus producing alterations in genome-wide methylation [103].

LncRNAs associated with DNA methylation machinery driving gene dysregulation are found in promoters of miRNAs, as well as of tumor suppressors.

Antisense of IGF 2 receptor (IGF2R) non-protein coding RNA (AIRN) functions in cis on *IGF2R* gene, causing steric hindrance and blocking Pol II binding at the transcription start site of *IGF2R*, consequently, *IGF2R* promoter is methylated and the gene is silenced [101, 102].

## LncRNAs involved in gene activation

LncRNAs regulate gene transcription by scaffolding and tethering enzyme complexes on the target gene promoters, and assembly promotes demethylation, chromatin decondensation, and recruitment of transcription factors [104]. Transcription activating lncRNAs recruit DNA methyltransferases DNMT1 and DNMT3B at gene promoter sites and promote the binding of WDR5 and MLL complexes, resulting in elevated levels of H3K4me3. Some RBPs such as FBL, EIF4A3, UPF1, WDR5, and YTHDF1/2/3 act as mediators of lncRNA action on protein-coding genes. WDR5, a subunit of the chromatin remodeling complex MLL1, has RBP activity, containing an RNA-binding pocket that interacts with lncRNAs and regulates their target genes. WDR5 can be attracted to a gene promoter by lncRNAs to demethylate the promoter, modify the histone marks, and activate target gene expression.

TCF21 antisense RNA inducing demethylation (TARID) activates the expression of TCF21 through promoter demethylation. TARID interacts with the TCF21 promoter as well as with GADD45A, regulating DNA demethylation. GADD45A can recruit thymine-DNA glycosylase that exerts base excision repair-mediated demethylation, involving oxidation of 5-mC to 5hmC in the TCF21 promoter by TET methylcytosine dioxygenases [105].

A series of lncRNAs mediating activation of gene expression through histone marks or enhancer and chromatin looping is provided in Table 3.

**Table 3:** Gene activation by lncRNAs through euchromatin regulation and histone marks

**Gene**	**Interaction**	**Target**	**Histone mark**	**Reference**
*GClnc1*	WDR5, KAT2A	SOD2 promoter	H3K4me3, H3K9ac	[84, 88]
*GCAWKR*	WDR5, KAT2A	PTP4A1	H3K4me3, H3K9ac	[85]
*TM4SF19-AS1*	WDR5	TM4SF19	H3K4me3	[84]
*LincRNA-P21*	hnRNPK	P21	H3K4me3, H3K9ac	[89]
*Gm29348*	-	-	H3K4me3, H3K9ac	[90]
*TARID*	GADD45A	TCF21	CpG	[105]
*lncPRESS1*	SIRT6	Embryogenesis	H3K56ac, H3K9ac	[106]
*HOTTIP*	WDR5	HOXA	H3K4me3	[107–109]
*CCAT1-L, PVT1*	CTCF, hnRNPK	MYC	Chromatin loop, NUP153 binding to cohesin	[110]
*NRIP enhancer*	Cohesin	ER dependent genes	Chromatin loop nuclear receptor interacting protein	[111, 112]
*CHASERR*	CHD2	CHD2 regulated promoters	Binding to nascent RNA	[44, 113]
*HASTER*	-	HNF1A	Promoter enhancer interaction	[114]
*NEAT1_2*	RBP14	Euchromatin binding	Paraspeckle condensates	[115]
*THRIL*	hnRNPs	TNF-α	Promoter/enhancer activation	[44, 101]
*HOTAIR*	LSD1	HBXIP-MYC dimer MYC target genes	Lysine demethylation	[44]
*NeST*	WDR5	IFN-gamma	H3K4me	[116]
*Lnc13*	hnRNPD	Suppress Inflammatory genes by chromatin access	Celiac disease	[12]

CCAT1-L: Colon cancer associated transcript 1-long; PVT1: plasmacytoma variant translocation 1; CHASERR: CHD2 adjacent suppressive regulatory RNA; MYC: viral myelocytomatosis homolog; LSD1: lysine-specific demethylase 1; NEAT1: nuclear enriched abundant transcript 1; GADD45A: growth arrest and DNA-damage-inducible alpha; CTCF: CCCTC-binding factor; CHD2: chromodomain-helicase DNA binding protein 2; -: blank cell

Another RNA, lincPRESS1, interacts with and sequesters SIRT6 (an HDAC and NAD dependent sirtuin), and this inhibition preserves the acetylation of histone H3 (H3K56ac and H3K9ac) in euchromatin favoring the transcription in embryonic stem cells [106].

HOTTIP binds the WDR5 protein and guides the MLL/SET1 histone MTC, resulting in H3K4me3 marks and activation of *HOXA* genes [107–109].

Enhancer and promoter regions are transcribed into eRNAs and promoter upstream transcripts [109]. A super-enhancer lncRNA, CCAT1-L, induces chromatin interactions between *MYC* enhancers and promoters: CCAT1-L recruits the DNA-binding protein CTCF, which consequently activates *MYC* expression: the 5’ end of CCAT1-L interacts with hnRNPK ribonucleoproteins, and both interact with the MYC promoter and with PVT1 RNA to orchestrate their expression [110].

Another example of chromatin rearrangement is provided by NRIP enhancer: NRIPe recruits nuclear proteins such as cohesins, promoting chromatin looping and activating genes in short and long distances, and upon activation of the estrogen receptor regulates transcription [111, 112].

CHASERR is localized upstream of the chromatin remodeller CHD2. CHD2 binds nascent RNAs, including CHASERR, and promotes gene expression. The reciprocal regulation of CHD2 and its lncRNA is a regulatory feedback loop, in which CHD2 regulates its own expression and that of CHD2 regulated promoters [44, 113].

Close to the gene coding for HNF1A coding for a homeodomain transcription factor, is an antisense lncRNA, HASTER: its promoter modulates HNF1A promoter-enhancer interactions in cis, and in this way activates HNF1A transcription [114].

NEAT1 has several similarities shared with metastasis-associated lung adenocarcinoma transcript 1 (MALAT1): the first one is present in paraspeckles, the second in nuclear speckles and is also called NEAT2. NEAT1 binds to active chromatin sites, as by data of Capture Hybridization Analysis of RNA Targets (CHART), and should have a role in transcription regulation. The 23 kb isoform NEAT1_2 is necessary for the formation of nuclear paraspeckles, in which membrane-less condensates form a gel like phase separation similarly to HP1 aggregates within chromatin regions: NEAT1_2 dependent condensates affect the sequestration of proteins like fused in sarcoma (FUS), that aggregates around RNA, together with the RNA binding protein RBP14, non-POU domain-containing octamer-binding protein (NONO), which interacts with paraspeckle protein component 1 (PSPC1), splicing factor proline glutamine rich SFPQ, the Drosophila behavior/human splicing (DBHS) protein family are the core components of paraspeckle, in addition to almost 60 other additional proteins: a role for this transcription activating complexes is the activation of specific genes like IGF1R; PSPC1, in addition to the role in paraspekle complexes, has its own role by inhibiting the RNA-induced premature release of Pol II; mTOR negatively regulates NEAT transcription, suppresses paraspeckles and frees NONO, SFPQ and RBP14, involved in RNA splicing [115].

## LncRNAs regulated at epitranscriptomic level

The most diffuse modifications in RNAs are m6A and m5C [12], while m7G are rare; m1A modifications in MALAT1 and N4 acetylcytidine (ac4C) modifications have also been studied [117–119].

There are eight RNA m5C-methyltransferases belonging to two families: the NSUN family containing few isoforms (NSUN1 to NSUN7) and DNMT2 family members, which are m5C writers. Two m5C readers have been identified: the ALYREF and the YBX1. No information is known for m5C erasers but this role can be ascribed to TET enzymes [12].

The methyltransferases METTL3 and METTL14 are writer proteins that methylate adenine to m6A in RNAs; METTL14 is a chromatin regulator independent of its RNA m6A methyltransferase activity [120]. METTL3 facilitates METTL14 catalytic activity, through dimerizing together. METTL3 contains SAM, therefore is dependent on SAM availability.

One reader of m6A modification is the IGF2BP family, which regulates the stability of m6A modified mRNAs; additional m6A readers are eIF3, these two known as RBPs; the hnRNP family and proteins containing the YTH domain: members of the YTH domain family are YTHDF1 and YTHDF2. Several of these reader proteins have RNA binding activity.

The eraser enzymes are FTO, an RNA demethylase, possibly acting on N6,2’-O-dimethyladenosine (m6Am), and the alkylation repair ALKBH5, an α-KG-dependent dioxygenase [1]. The cytoplasmic protein YTHDF2 specifically recognizes and destroys m6A-modified RNAs to facilitate the translocation of bound mRNAs and promotes mRNA translation through its interaction with translation initiation factors. In nuclei, the YTHDC1 protein regulates pre-mRNA splicing by recruiting and regulating splicing factors.

METTL14 colocalizes with chromatin with repressive H3K27me3 marks. METTL14, but not METTL3, binds H3K27me3 and recruits KDM6B to induce H3K27me3 demethylation independent of METTL3, and this activity is required in the passage from embryonic stem cells to differentiated cells [120].

The deposition of the m6A mark induces structural changes in lncRNAs, thus modifying lncRNA-protein-interactions. Furthermore, the m6A modification modulates gene transcription, influencing the subcellular localization of lncRNAs and regulating lncRNAs stability.

HOTAIR transcript can contain m5C and m6A marks. Other lncRNAs with several epigenetic marks are MALAT1 (with three m6A and seven m5C), TUG1, GAS5, lincRNA1281, TERC, RP11, CASC11, NEAT1, SRA1, ANRIL (with six m6A modifications) and SNHG members. METTL14-mediated m6A inhibits the expression of Xist through YTHDF2-dependent RNA degradation [84]. The abundance of lncRNAs is directly correlated with levels of SAM and MAT2A [1]. It has been shown that m6A modifications regulate mRNA stability [121].

There is a correlation between m6A modifications and lncRNAs levels. In particular, lncRNAs may influence the stability and degradation of enzymes involved in m6A deposition, and facilitate their integration into protein complexes. FEZF1-AS1, whose silencing in multiple myeloma leads to increased cell death by regulation of IGF2BP1, an m6A reader [86]. DARS-AS1 promotes the recruitment of METTL3 and METTL14 to DARS mRNA to induce m6A modification and enhance translation in cervical cancer [122]. THOR transcripts, which contain m6A marks, recruit m6A readers YTHDF1 and YTHDF2 that regulate THOR stability and decay. These m6A-dependent RNA-protein interactions can maintain the oncogenic role of THOR, whose stability leads to the proliferation and invasive phenotype of cancer cells [123]. LNCAROD is highly expressed and induces a malignant phenotype of HNSCC, with METTL3- and METTL14-mediated m6A modifications enhancing LNCAROD stability. LNCAROD facilitates YBX1 and HSPA1A interaction, protecting YBX1 from degradation [124]. GAS5 is an antioncogene that blocks CRC progression by interaction with YAP, promoting YAP phosphorylation and degradation. GAS5 is negatively regulated by the m6A reader YTHDF3: m6A modification sites on lincRNA-GAS5 are recognized by YTHDF3, leading to GAS5 degradation and promoting cancer progression [125]. In NSCLC cells, METTL14-induced m6A modification reduces LINC02747 levels through m6A “reader/eraser” YTHDC2 leading to RNA degradation: LINC02747 activates CDK4/CyclinD1 complex and PI3K/AKT pathway [126].

Two RBPs, IGF2BP2 and YTHDF1, have been identified as responsible for establishing m6A marks on CDC6 mRNA and DLGAP1-AS2, and this contributed to their stabilization [29].

LINC021 oncogene promotes CRC proliferation by interacting with IGF2BP2 and enhances MSX1 and JARID2 mRNA stability [127]. LINCRIS (LINC00920) prevents K139 ubiquitination and degradation of m6A reader protein IGF2BP2, defining a role for the LINRIS-IGF2BP2-MYC axis in CRC progression [127]. IGF2BP2 interacts with differentiation antagonizing non-protein coding RNA (DANCR) marked by m6A modification and increases DANCR expression in pancreatic cancer (PC) cells. This lncRNA is involved in HCC, osteosarcoma, and acute myeloid leukemia. DANCR is modified with m6A, so that IGF2BP2 binding to m6A-modified DANCR stabilizes DANCR RNA [127]. METTL3 increases m6A modifications in LINC00958 and inhibits lncRNA degradation in HCC [127].

KCNK15-AS1 is down-regulated in PC tissues. ALKBH5 is required to induce m6A demethylation of KCNK15-AS1 and to favor KCNK15-AS1 up-regulation, required for PTEN induction by interaction with MDM2 to degrade REST by ubiquitination [128]. In hepatocellular cancer, CASC11 associates with ubiquitin-conjugating enzyme E2T (UBE2T) mRNA and stabilizes it. CASC11 decreases UBE2T m6A levels through ALKBH5 recruitment. In addition, CASC11 inhibits the association between UBE2T mRNA and m6A reader protein YTHDF2 [129].

KIAA1429 is a component of the m6A MTC. GATA3-AS enhances the interaction of KIAA1429 with GATA3 pre-mRNA as a molecular scaffold, the 6mA mark attracts the RNA-binding protein HuR leading to GATA3 degradation in HCC cells [127]. LINC0047 was shown to recruit the m6A writer METTL3 and to increase m6A modification on PTEN, decreasing PTEN stability, and inducing gastric cancer [127]. ARHGAP5-AS1 stimulates m6A modification of ARHGAP5 mRNA to stabilize it, by recruitment of the m6A writer METTL3 in the cytoplasm, and this event induces gastric cancer chemoresistance [127]. NEAT1_1 and NEAT1_2, are RNA enriched in paraspeckles, and m6A modified at several positions. ALKBH5 eliminates the m6A modification of NEAT1_2, influencing the expression of EZH2 and increasing the progression of gastric cancer [130]. METTL14 decreases the expression of NEAT1_1 in an m6A-dependent manner, followed by the action of the eraser YTHDF2 that leads to NEAT1_1 degradation [131].

In esophageal squamous cell cancer (ESCC) cells, LINC00022 interacts with the p21 protein and enhances the ubiquitination-mediated degradation of p21. The m6A eraser FTO reduces LINC00022 m6A methylation levels, resulting in the inhibition of RNA decay, regulated by the m6A reader YTHDF2. In ESCC cancer, FTO drives LINC00022-dependent ESCC cell proliferation [127]. FTO and ALKBH5, by erasing the m6A marks in LINC00022 the first one, and NEAT and KCNK15-AS1 the second, affect the stability and lead to the degradation of these lncRNAs [127]. PVT1 promotes cell proliferation and cancer stem-cell-like properties of HCC by binding to and stabilizing the RNA-binding protein NOP2. In ovarian cancer, PVT1 has various m6A marks: when high PVT1 levels correlate with cancer, the m6A marks are found low. By silencing ALKBH5, a decrease in PVT1 RNA levels was observed [132].

CHASERR is overexpressed in glioma tissues, and the m6A mark deposited by METTL3/YTHDF1 is abundant and correlated with overexpression [133]. LEAWBIH (lncRNA epigenetically activating Wnt/β-catenin signaling in HCC) contains m6A marks that allow its binding by the m6A reader YTHDC1, followed by interaction with and recruitment of H3K9me2 demethylase KDM3B to the *CTNNB1* promoter, leading to *CTNNB1* transcription, thus activating Wnt/β-catenin signaling [134].

FTO-IT1 is correlated to the proliferation and glycolysis of HCC cells and contributes to HCC malignant phenotype by the increase in glycolysis. FTO-IT1 recruits ILF2/ILF3 protein complex to 3’UTR of FTO mRNA, induced the stabilization of FTO mRNA, translated into the FTO protein, eraser of m6A. FTO decreased m6A modification on mRNAs of the glycolytic genes GLUT1, PKM2, and c-MYC, which reduced YTHDF2-mediated mRNA degradation [135]. Transformer 2 alpha homolog (TRA2A), a serine/arginine-rich splicing factor, influences methylation of lncRNAs, in particular MALAT1, in esophageal carcinoma, by forming a complex with METTL14, RBMX, and KIAA1429, another m6A writers [136].

In PC, FTO participates in upregulating LINC01134, acting on LINC01134 mRNA stability through YTHDF2. Higher expression of LINC01134 in PC was linked to increased m6A marks in LINC01134 and correlated well to the levels of expression of FTO and YTHDF2. FTO erases the m6A marks while YTHDF2 mediates the degradation of m6A-modified RNA, therefore LINC01134 devoid of m6A marks remains more stable in PC cells [137]. A relationship between the abundance of lncRNAs and the levels of SAM, including the SAM producing enzymes, such as MAT2A, has been demonstrated [91]. Therefore, m6A writers may change their activity depending on SAM availability. A schematic synthesis of the data on epitranscriptomic effects in lncRNAs is presented in Table 4.

**Table 4:** LncRNAs and the m6A modification (interaction with writers, readers, erasers)

**LncRNA**	**Interaction**	**Modified**	**Effect**	**Reference**
FEZF1-AS1	IGF2BP1 sequestration	m6A	Apoptosis	[121]
DARS-AS1	METTL3-METTL14	m6A	Proliferation	[86]
THOR	Readers YTHDF1, YTHDF2	m6A	Oncogenesis	[122]
LNCAROD	METTL3-METTL14	m6A	Prevents YBX1 degradation	[123]
GAS5	Reader YTHDF3	m6A	GAS5 degradation	[124]
LINC02747	Reader YTHDF3	m6A	GAS5 degradation	[125]
LINC021	IGF2BP2	m6A	MSX1 stability	[126]
LINCRIS	Stabilization of IGF2BP2	m6A	Prevents K139 ubiquitination	[127]
DANCR	IGF2BP2 stabilizes the mRNA	m6A	-	[127]
LINC00958	METTL3 stabilizes mRNA	m6A	Inhibits degradation	[126]
KCNK15-AS1 downregulated	ALKBH5 eraser	m6A	Stabilization	[127]
LINC0047	METTL3	m6A	PTEN-m6A degradation	[126]
CASC11	ALKBH5	m6A	Decrease m6A UBE2T	[129]
CASC11	YTHDF2	m6A	Inhibits the association between UBE2T mRNA and m6A reader protein YTHDF2	[129]
ARHGAP5-AS1	METTL3	m6A	Stabilized by m6A	[126]
NEAT1_2	ALKBH5, YTHDF2	m6A	Destabilize mRNA	[130]
LINC00022	FTO	m6A	Block degradation by YTHDF2.	[126]
PVT1	Silencing ALKBH5	m6A	Decrease levels	[131]
CHASERR	METTL3/YTHDF1	m6A	Overexpressed	[132]
LEAWBIH	YTHDC1	m6A	Recruits H3K9me2 demethylase KDM3B	[133]
FTO-IT1	Recruits ILF2/ILF3 protein complex to 3’UTR of FTO mRNA, induced FTO stabilization	m6A	Decreased m6A modification on GLUT1, PKM2, c-MYC, alleviating YTHDF2-mediated mRNA degradation	[134]
MALAT1	Serine/arginine-rich splicing factor TRA2A forms a complex with writers METTL14, RBMX, KIAA1429	m6A	lncRNA m6A methylation	[135]

CASC11: Cancer susceptibility 11; CHASERR: CHD2 adjacent suppressive regulatory RNA; MALAT1: metastasis-associated lung adenocarcinoma transcript 1; PVT1: plasmacytoma variant translocation 1; IGF2BP1: insulin growth factor 2 mRNA binding protein 1; METTL3: methyltransferase-like 3; YTHDF1: YT521-B homology domain family 1; ALKBH5: AlkB homolog 5; FTO: fat mass- and obesity-associated protein; TRA2A: transformer 2 alpha homolog; YBX1: Y-box-binding protein 1; PTEN: phosphatase and tensin homolog; UBE2T: ubiquitin-conjugating enzyme E2T; -: blank cell

NAT10 is the enzyme writer of ac4C, a novel epitranscriptomic event involved in several cancers. The role of NAT10 as a metastasis suppressor has been identified in CRC. NAT10 induces ac4C modification of several targets such as the lncRNA CTC-490G23.2 that interacts with polypyrimidine tract-binding protein 1 (PTBP1) to increase CD44 alternative splicing in primary ESCC: the lncRNA acts as a scaffold to increase the binding of CD44 pre-mRNA to PTBP1, resulting in an oncogenic splicing switch from the standard isoform CD44s to the variant isoform CD44v(8-10) [117]. NAT10 may behave also as a metastasis promoter and EMT inducer by targeting COL5A1 mRNA by increasing its stability [118]. m6A, m5C, m7G, and m1A modifications have been collected in useful databases, together with information about the enzymes responsible for writing and erasing them [137–141]. In particular, a link has been observed for m6A addition and cisplatin resistance in NSCLC. Han et al. [142] have found an important role of internal N7-methylguanosine among lncRNA modifications, relevant in resistant acute myeloid leukemia. Concerning internal m7G writers, reports indicate the involvement of METTL1 in association with the WDR4 protein complex, active on tRNAs, rRNA, mRNAs (improving mRNA translation), and probably miRNAs. Information about readers and erasers is still lacking. One database (http://www.xjtlu.edu.cn/biologicalsciences/m7ghub) is proposed here as a useful tool to check updates on this topic. Twelve m6A/m5C/m1A/m7G-associated lncRNAs (AL080276.2, AC092111.1, SOX21-AS1, DNAJC9- AS1, AC025171.1, AL356019.2, AC017104.1, AC099850.3, UNC5BAS1, C006064.2, AC010319.4, and AC016822.1) have been linked to prognosis of cancers such as glioma. Butler and Banday [143] have focused on adenine deamination by APOBEC3 and the consequent mutations leading to cancer and proposed therapeutic approaches to restore RNAs involved in tumor control.

## Scaffolding RNAs involved in DNA stability

A last group of lncRNAs involved in assembling protein complexes and RBPs. These lncRNAs do not have a direct role in epigenetic control or epitranscriptomic regulation of RNA but are relevant in cancer development for their role in DNA stability and DNA repair. The prototype of this class is represented by ncRNA activated by DNA damage (NORAD). NORAD is a ubiquitously expressed cytoplasmic ncRNA that is activated by DNA damage. NORAD has a role in regulating the assembly of the RBMX-dependent ribonucleoprotein complex, which contains topoisomerase TOP1. Furthermore, NORAD interacts with several subunits of the MCM2-7 replication helicase, which plays a central role in DNA replication initiation and elongation [144]. The main interacting proteins are Pumilio1 (PUM1) and PUM2, through Pumilio recognition elements (PREs), consisting of five repetitive ~400 nucleotide (nt) domains known as NORAD domains (ND1–ND5), and in this way complexes and sponges PUM1 proteins, responsible of translation repression, into NORAD-Pumilio (NP) bodies. The ND4 domain is the most conserved and was shown to bind proteins such as PUM1, SAM68, IGF2BP2/3, PABPN1, and RBMX [144]. NORAD preserves normal mitosis by binding and inhibiting Pumilio proteins that, if hyperactivated, can induce chromosome instability. Noteworthy IGFBP2/3 are relevant for the role in reading m6A marks, promoting m6A-modified mRNAs more stable. This activity makes NORAD entitled for mention in this review referring to scaffolding lncRNAs related to epigenetic mechanisms.

Among the m6A associated proteins driving writer enzyme METTL3 to target RNAs, are TRA2A and CAPRIN1, two RBPs that confer specificity to writers [145]. CAPRIN1 is a phase-separating protein, found relevant in dendritic spines and in the setup of autism, and associated with stress granules and with a variety of ncRNAs [146, 147].

Among the lncRNAs interacting with protein complexes, they exert two important roles, the first in the response of p53 protein, the guardian of the genome [34], and the second, in the response to DNA damage, in homologous recombination (HR), and non-homologous end joining (NHEJ). These pathways are relevant for cancer therapies with Poly ADP-ribose polymerase inhibitors (PARPi) when effective, especially in BRCA1^–/–^ mutants or in synthetically lethal cells. In this paragraph a mention will be given to LINP1 RNA, with a role in the scaffolding of multiple Ku70-Ku80 dimers and DNA-PKs at DNA damage sites; LINP1 forms multiprotein complexes with RPA, Ku70, Ku80, and ISWI chromatin remodeler, that bring together BRCA1, 53BP1, CDH4, and PARP1 [148]. A scaffolding role was proposed for NIHCOLE RNA, whereby its interaction with several Ku80 units, promotes the formation of multimeric NHEJ complexes to increase ligation efficiency by promoting phase separation to drive NHEJ via DNA repair “hubs” [148]. NIHCOLE supports multimeric repair complexes including the ligation complex formed by XRCC4 and DNA ligase IV. Another lncRNA, LRIK, was also found to interact with the Ku70-Ku80 heterodimer. ChIP of cells with induced DSBs showed a requirement of LRIK binding to damaged DNA in the accumulation of Ku heterodimers at DSB sites, and the recruitment of DNA-PKcs and XRCC4 [148]. BS-DRL1 RNA also interacts with HMGB1 and facilitates its assembly on chromatin upon DNA damage. Aerrie RNA associates with YBX1 and is recruited to damage sites to enhance DNA repair [148]. MALAT1 is enriched in nuclear speckles. MALAT1 through the binding with PARP1/LIG3 complex is involved in the alternative NHEJ (A-NHEJ) pathway. Anti-MALAT1 therapy was effective in vitro using antisense oligonucleotides (ASOs), and RNase H degradation using antisense gapmer DNA oligos [149].

DNA damage-sensitive RNA1 (DDSR1), a 1,600 nucleotide transcript, is induced upon DSB in DDR and regulates BRCA1 activity and 80 localization at DSB sites [150].

SNHG12 stabilizes the interaction between DNA-PK and Ku70/Ku80 and supports NHEJ based repair of DNA.

Lastly, a group of ncRNAs found important in DDR exerts a role as decoys, by protecting target proteins from undesired interactions. For instance, HITT RNA binds to ATM, preventing the formation of the MRE11-RAD50-NBS1 (MRN) complex relevant in HR and in NHEJ [151]. The lncRNAs involved in the DDR are not treated further in this review. Interestingly, a micropeptide originating from the RNA CTD-2256P15.2, named Poly ADP-ribose (PAR)-amplifying and CtIP (CTBP interacting protein)-maintaining micropeptide (PACMP), is required for efficient PARylation by PAR polymerase enzymes. Targeting the micropeptide alleviates resistance to several chemotherapeutics.

PARPi are currently applied in the therapy of various cancers and may exploit new synthetic lethal phenotypes derived from the silencing of DNA damage-induced lncRNAs. As an example, MALAT1 in prostate cancer has been knocked down by ASOs technology: the effect was a malfunction of homology recombination either in HR deficient and HR proficient cells [152]. As for H19, its depletion abrogates DNA repair and provides sensitivity to PARPi therapy. H19 increases the stability of BRCA1 by interfering with the proteasomal degradation master proteins ubiquitin ligases HUWE1 and UBE2T [153].

## Progress in lncRNAs studies and therapeutics

The knowledge on RNA regulators and the epitranscriptome activities of enzymes modifying mRNAs and lncRNAs has been translated into therapeutic approaches to restore perturbations in the writers-readers-erasers of RNA modifications, that show a peculiar profile depending on cancer type. Novel databases have been set up to collect data on lncRNAs involved in epigenetic regulation [154, 155].

The world of RNA modifications and the role that these modifications have on RNA metabolism and stability is also a field of interest in cancer therapeutics.

One suppressor lncRNA, linc-SPRY3-2/3/4, was found to mediate IGF2BP3 activity and to increase the response to radiotherapy on the Y chromosome [156]. FEZF1-AS1 may be silenced in multiple myeloma in order to increase cellular apoptosis by regulation of the m6A reader protein IGF2BP1 [157].

UCA1 RNA colocalizes with METTL14 in nuclei and cytoplasm, and the two can be pulled down together. UCA1 stabilizes METTL14 allowing the writer to deposit m6A marks on target mRNAs, such as CYP1B1 and CXCR4, leading to acute myeloid leukemia (AML) development [158].

CASC9 RNA is highly expressed in glioblastoma in its m6A modified form: m6A reader IGF2BP2 stabilizes CASC9 RNA, so that CASC9 may increase hexokinase 2 (HK2) mRNA stability and consequently HK2 activity [159].

The understanding of lncRNAs and their mechanisms of interaction with epigenetic enzymes pushed for the development of lncRNA-based therapeutic targeting through ASOs and CRISPR/Cas technology [109].

SAM levels influence metabolic enzymes whose mRNAs are affected by m6A marks. Therefore, the SAM biosynthesis pathway has been targeted by inhibitors, improving multiple myeloma conditions [160]. The small molecule compound FIDAS-5 targeting MAT2A reduced tumor size in 5TGM1 murine cells. It was found that MAT2A inhibition can synergistically enhance the anti-MM effect of bortezomib [161].

Since epitranscriptomic modifications of lncRNAs affect their stability and function in cancer, several groups targeted writers, readers, and erasers of these modifications. As for METTL3A inhibition, this target has been selected for cancers with high m6A levels, and inhibitors have been already tested in clinical trials, such as METTL3 inhibitor STC-15, developed by storm therapeutics [162, 163]. An m6A inhibitor (STM-2457) targeting METTL3 has entered phase I clinical trials in 2022, showing that the drug inhibits the proliferation of AML. Similarly, other inhibitors targeting enzymes responsible for adding and removing m6A mark (METTL and FTO proteins, respectively) have entered phase 1 clinical trials for AML in 2022 [164, 165].

Huang et al. [166] developed two potent FTO inhibitors, FB23 and FB23-2, two tricyclic benzoic acids, which directly bind FTO and selectively block its m6A demethylase activity, significantly inhibiting the proliferation of AML cell lines and primary maternal AML cells.

Later, they discovered that FTO inhibitors CS1 and CS2 can inhibit the self-renewal of cancer stem cells and enhance T cell toxicity [167]. The inhibition of FTO demethylase with the small inhibitor Dac51 significantly decreased the leiomyosarcoma cell proliferation in a dose-dependent way, by means of cell cycle arrest [168, 169]. However, the tumor-promoting and suppressive roles of FTO in different cancer types must be taken into account [169].

Another m6A mark reader has been studied for a therapeutic approach, using vesicle-like nanoparticles (VNPs)-encapsulated YTHDF1-siRNA for YTHDF1 silencing in vivo [170].

Several cancers are progressing through chromosomal instability (CIN). Therapeutic strategies may be designed to restore radio- and chemotherapy response via the CIN pathway. The cancer cell cannot tolerate too much CIN; hence, one can accelerate CIN pathways and generate less-fit karyotypes. The other option is to inhibit CIN and therapeutically tackle a stable and genetically frozen cancer cell population [171].

In breast cancers of various subtypes low m6A levels were found, depending on the downregulation of m6A methylases, so the expression and activity of m6A erasers (FTO, ALKBH5) have a small influence on mRNA methylation levels [172]. Application of small molecule regulators and inhibitors of m6A metabolism, such as R-2hydroxyglutarate, cannot be applied in all cases [173]. In cancer types with low m6A levels, such as breast cancers, m6A levels are low, so erasers have relative roles in growth control; instead, m6A writers such as METTL3A can be targeted by small activators.

As for demethylases, FTO and ALKBH5, belonging to the 2-oxoglutarate (2-OG) dioxygenase class, progress has been made for FTO using several classes of inhibitors, such as metal-chelating inhibitors, substrate competing inhibitors, and scaffolds with different structure; while, for ALKBH5, 2-oxoglutarate-type inhibitors show good efficacy [1, 174].

As discussed, 2HG is an inhibitor of FTO N6-methyladenine demethylase activity, when this eraser enzyme is blocked, this increases m6A levels. In addition, citrate inhibits α-KG dependent N6-methyladenine demethylase ALKBH5 [1]; as for SAM synthetase, it was shown that oxidized glutathione GSSG inhibits SAM synthetase. Depending on the type of cancer and of lncRNA targeted, m6A writers may be selected for inhibition by small therapeutic compounds [174–178].

Pharma companies succeeded in the development of novel compounds targeting epigenetic enzymes [176], such as HDAC inhibitors which are approved for T cell lymphoma: belinostat [177], vorinostat [178], romidepsin [179], and chidamide [180], and multiple myeloma (panobinostat) [181].

In addition, hematologic malignancies, particularly AML [182, 183], have found effective treatments in 5-azacitidine [184], decitabine [185], and guadecitabine [186] that target DNMTs. Furthermore, for m5C methylase, azacitidine, and decitabine are cytidine analogs that inhibit any m5C methylase and have been approved for clinical use in hematological malignancies [185–187]. Studies using a CRISPRa approach and optogenetic tools showed the ability to functionalize the role of lncRNAs in drug resistance [188, 189]. Ongoing oncological clinical trials go beyond epigenetic enzymes, and focus on emerging targets such as bromodomains for multiple myeloma, solid tumors, and lymphoma, while new advancements are in progress on understanding RNAs function inside the cells [190–194].

EZH2 is overexpressed in lymphoma. Tazemetostat that inhibits the HKMT EZH2 was approved for epithelioid sarcoma [195]. Novel inhibitors have been developed to block EZH2 in these cancers [196, 197]. Several studies were focused on EZH2 inhibition, in order to improve chromatin accessibility and H3K27 demethylation: in the attempt to block the interaction between HOTAIR and the PRC2 complex, small sequences targeting a candidate interaction binding domain was tested (AC1NOD4Q) blocking the interaction domain in EZH2 [198, 199]. AC1Q3QWB was found to disrupts PRC2 recruitment to chromatin, enhancing the antitumor effect of 3-deazaneplanocin (DZNep), functioning as SAHH inhibitor, and consequently inhibiting histone methyltransferases (HMTs) [200]. In other types of tumors, lncRNAs-EZH2 interaction was selected as a promising therapeutic target, such as in cutaneous melanoma. Novel compounds are being developed to interact with regulatory domains or active sites, to target under-functioning or malfunctioning enzymes.

It is worth noting that He et al. [201] identified exon junction complexes as m6A suppressors, which could inhibit the m6A methylation of exon junction-proximal RNA within coding sequences. There are various other proteins and enzyme co-operators that may show important anticancer effects when blocked by inhibitors. For instance, the complex formed by METTL3–METTL14 is worth being targeted by activating compounds. In particular, METTL14, aside from interacting with METTL3, exerts specific roles in transcription activation [202, 203].

### LncRNAs conferring resistance to drug inhibitors

One focus of pharma research is on the identification of cooperativity between regulatory RNAs and survival factors in cancer cells. For instance, by using libraries of oligonucleotides, single guide RNAs (sgRNAs) and CRISPR/Cas technology [204], lentivirus vectors for cell transformation, researchers found which lncRNAs were responsible for chemoresistance and for escape of kinase inhibitor treatment. The study allowed to identify lncRNAs that cooperated in addition to v-Raf murine sarcoma viral oncogene homolog B (BRAF) mutations to establish resistance to BRAF inhibitor in melanoma cells: these lncRNAs overexpressed in BRAFi-resistant cell lines have been determined to contribute to a gain-of-function phenotype [205, 206]. Proteolysis targeting chimeras (PROTACs) is a novel strategy to knock down proteins of interest, showing efficiency at nanomolar levels on BRAFi-resistant cell lines and advantages over conventional small molecule inhibitors [205]. Finally, the emergence of small molecules as efficient RNA binders [207–210], together with technological advances, provide a timely start for novel approaches targeting epigenetic modifications. Recently, light-dependent control of regulatory RNA allowed to activation of an RNA probe, synthetic genes, and short regulatory RNAs, as well as miRNA mimics, in a spatiotemporal manner, for evaluating their effects inside the cell [211].

The need to understand lncRNAs function and their mechanisms of interaction with epigenetic enzymes pushed up for the development of lncRNA-based therapeutic targeting, exploiting RNA silencing, ASOs, and CRISPR/Cas technology [109, 190].

### Mechanism of therapy resistance mediated by lncRNAs

Several cancers are progressing through chromosomal instability (CIN). Therapeutic strategies may be designed to restore radio- and chemotherapy response via the CIN pathway. The cancer cell cannot tolerate too much CIN; hence, one can accelerate CIN pathways and generate less-fit karyotypes. The other option is to inhibit CIN and therapeutically tackle a stable and genetically frozen cancer cell population.

In CRC patients, the lncRNA *Xist* was upregulated in patients who showed no response to 5-FU compared to those who showed a response by inducing thymidylate synthase.

In breast cancer cells, forced expression of *H19* promotes DNA damage repair and resistance to PARP inhibition, whereas *H19* depletion diminishes DNA damage repair and increases sensitivity to PARP inhibitors. *H19* exerted its functional roles via direct interaction with ILF2 in the cell nucleus. *H19* and ILF2 increased BRCA1 stability via the ubiquitin-proteasome proteolytic pathway via the *H19*- and ILF2-regulated BRCA1 ubiquitin ligases HUWE1 and UBE2T [153].

Targeting MALAT1 in association with PARPi: in triple negative breast cancer, lncRNAs can be used to cluster the patients in subgroups, while five transcripts of the MALAT1 gene are specifically upregulated in resistant patients and are involved in resistance to chemotherapy [148, 151–153, 211].

In gastric cancer, ABL is involved in the antiapoptotic pathway, by interaction with APAF1 and inhibition of APAF1 recognition by cytochrome c; targeting ABL may restore the apoptosome and caspase activation leading to cell death [85, 212].

Several publications approached the topic of targeting lncRNAs for cancer treatment [212–216] also in the attempt to overcome chemotherapy resistance.

Small molecule ligands can be the base for backbones and scaffolds to provide modified skeletons able to interact with RNA structures in lncRNA: one case is the diphenylfuran (DPF) group of MALAT1 interactors, which bind to MALAT1 triple helix [217].

NEAT1 is upregulated in TNBC and correlates with cell growth, migration, and invasion. Moreover, it mediated cisplatin/taxol drug resistance, so that NEAT1 downregulation could sensitize cancer cells to cisplatin/taxol treatment [218, 219].

P53-dependent apoptosis modulator (*PDAM*) and cancer upregulated drug resistant (*CUDR*) are two drug resistance-associated lncRNAs whose effect is to block apoptosis, thus affecting cisplatin resistance in oligodendroglial tumors and bladder cancer, respectively. Prostate cancer gene expression marker 1 (PCGEM1) blocks apoptosis by suppressing caspase7 activation, leading to doxorubicin resistance in prostate cancer.

In gastrointestinal cancers, CCAT2, BOP1, and AURKB form an RNA-protein complex that pulls the chromosomes in all directions, giving rise to chaotic cell division. This pathway is associated with resistance to 5-fluorouracil and oxaliplatin [220]. UCA1 and PD-1 proteins were silenced in mouse tumors, and the combined knockout decreased the tumor burden and prolonged overall survival by modulating the T cell-mediated immune response [221].

LncRNA inhibition includes the application of antisense anti-oligonucleotides (ASOs), siRNAs, short hairpin RNAs (shRNAs), CRISPR/Cas9-based genome editing, and small molecule inhibitors of ncRNAs. CRISPR/Cas9-based editing approaches, delivering the Cas9 nuclease complexed with a synthetic guide RNA (gRNA) to precisely cut the target ncRNA [221]. Vaidya et al. [222] showed the efficacy of a TNBC therapy based on nanoparticle-mediated RNAi of the oncogenic lncRNA DANCR using tumor-targeting RGD-PEG-ECO/siDANCR nanoparticles. In other reports, several approaches have been described to treat epigenetic dysfunctions dependent on lncRNAs [223–228]. It was shown that single wall carbon nanotubes (SWCNTs) may deliver nucleic-acid drugs stably and efficiently with good tolerability and minimal toxicity. Up to now, only two drugs based on ASO technology have been tested for possible application in therapy, and most of them are antisense to protein coding genes (bcl-2, VEGF, AP1), while two drugs target small RNAs (anti-mir-155 in cutaneous T-cell lymphomas, and miR-122, Miravirsen, tested in primates for liver disease) and others are miRNA mimics [184]. LncRNAs are complex molecules that assemble within the same RNA sequence in various domains, as regions complementary to miRNAs to act as miRNA sponges, domains functioning as decoys, and in some cases also micropeptides. A big problem is to target the antisense drug to specific cells, and this has been made possible for the liver, exploiting antibodies targeting surface receptors, but in other cases silencing an RNA target can be beneficial in one cell type but detrimental in other cell types. This step is very challenging and it will require more research and advancements to reach an applicative use for human cancer treatment.

## Conclusions

It has been found that a complex architecture of lncRNA-mediated chromatin dynamics is present in various cancers. The review highlighted the potential to target either lncRNAs as well as epigenetic enzymes in the attempt to test new therapeutic approaches in cancer treatment.

In conclusion, new compounds and inhibitors targeting DNMTs, HATs, HDACs, and HMTs, as well as strategies with the combination of multiple compounds can be effective, in association with existing epigenetic therapeutic drugs and more new therapies to improve the treatment of deregulated epigenetic regulation of RNAs. Several research groups envisaged that knowledge of lncRNAs will allow the development of lncRNA-based therapeutic targeting, by means of ASO, and the development of CRISPR/Cas regulation of genes involved in RNA-epigenetics crosstalk.

## Abbreviations

5-mC/m5C: 5-methylcytosine
ABL: apoptotic protease-activating factor 1-binding long noncoding RNAs
ALKBH1: AlkB homolog 1
AML: acute myeloid leukemia
APAF1: apoptotic protease-activating factor 1
ASOs: antisense oligonucleotides
AURKB: aurora kinase B
BRAF: v-Raf murine sarcoma viral oncogene homolog B
BRCA1: breast cancer type 1 susceptibility protein
CASC11: cancer susceptibility 11
CCAT1-L: colon cancer associated transcript 1-long
CDKi: cyclin-dependent kinase inhibitor
CGIs: CpG islands
CHD2: chromodomain-helicase DNA binding protein 2
ChIP: chromatin immunoprecipitation
CIN: chromosomal instability
CRC: colorectal cancer
CTCF: CCCTC-binding factor
DDR: DNA damage response
DNMTs: DNA methyltransferases
DSB: double-strand break
EED: embryonic ectoderm development
eIF: eukaryotic initiation factor
ESCC: esophageal squamous cell cancer
EZH2: enhancer of zeste homolog 2
FTO: fat mass- and obesity-associated protein
GADD45A: growth arrest and DNA-damage-inducible alpha
H3K4me1: mono-methylation on histone 3 lysine 4
HCC: hepatocellular carcinoma
HDACs: histone deacetylases
HKMTs: histone lysine methyltransferases
HNF1A: hepatocyte nuclear factor 1 homeobox A
hnRNPs: heterogeneous nuclear ribonucleoproteins
HOTAIR: HOX transcript antisense intergenic RNA
HP2: heterochromatin protein 2
HR: homologous recombination
IGF2BP: insulin growth factor 2 mRNA binding protein
JARID2: Jumonji, AT rich interactive domain 2
KMT: lysine methyltransferase
KMT2: histone-lysine *N*-methyltransferase 2
lincRNAs: long intergenic non-coding RNAs
lncRNAs: long non-coding RNAs
LSD1: lysine-specific demethylase 1
m6A: N6-methyladenosine
m7G: 7-methylguanosine
MALAT1: metastasis-associated lung adenocarcinoma transcript 1
MAT2A: methionine adenosyl transferase 2A
METTL3: methyltransferase-like 3
MLL: mixed-lineage leukemia
MTC: methyltransferase complex
MYC: viral myelocytomatosis homolog
ncRNAs: non-coding RNAs
NEAT1: nuclear enriched abundant transcript 1
NHEJ: non-homologous end joining
NORAD: non-coding RNA activated by DNA damage
NSCLC: non small cell lung cancer
NSUN: NOL1/NOP2/SUN domain
PAR: Poly ADP-ribose
PARPi: Poly ADP-ribose polymerase inhibitors
PC: pancreatic cancer
PcG: Polycomb group
Pol II: RNA polymerase II
PRC1: Polycomb repressive complexes 1
PRMTs: protein arginine methyltransferases
PTEN: phosphatase and tensin homolog
PUM1: Pumilio1
PVT1: plasmacytoma variant translocation 1
RBM15: RNA-binding motif protein 15
RBP: RNA binding protein
REST: RE1-silencing transcription factor
RNA-IP: RNA-immunoprecipitation
SAH: *S*-adenosyl homocysteine
SAHH: *S*-adenosyl homocysteine hydrolase
SAM: *S*-adenosylmethionine
SNHG7: small nucleolar RNA host gene 7
snoRNAs: small nucleolar RNAs
SUZ12: suppressor of zeste 12
TARID: TCF21 antisense RNA inducing demethylation
TETs: ten-eleven translocation
TRA2A: transformer 2 alpha homolog
UBE2T: ubiquitin-conjugating enzyme E2T
UTRs: untranslated regions
WDR5: WD repeat domain 5
Xist: X-inactive speciﬁc transcript
YBX1: Y-box-binding protein 1
YTHDF2: YT521-B homology domain family 2
α-KG: α-ketoglutarate

## Declarations

### Copyright

© The Author(s) 2024.
